# Os Vesalianum Pedis in a Professional Badminton Player: A Case Report

**DOI:** 10.7759/cureus.68411

**Published:** 2024-09-01

**Authors:** Nikhil Roy Mohan, Dobson Dominic

**Affiliations:** 1 Department of Sports Medicine, Saveetha Medical College and Hospital, Saveetha Institute of Medical and Technical Sciences, Chennai, IND

**Keywords:** foot and ankle disability index (fadi), professional athlete, foot injuries, accessory ossicle, ovp

## Abstract

Os vesalianum pedis (OVP) is one of the exquisite accessory ossicles adjacent to the foot's fifth metatarsal base. Though most of the OVP cases are asymptomatic, only a handful of patients who presented with lateral foot pain have been reported. A 36-year-old professional badminton player presented with swelling and tenderness in the right foot at the metatarsal base. An initial radiographic examination revealed a pseudo-Jones fracture. As a result of persistent pain and concerns regarding OVP, a reassessment of the X-ray was conducted. The examination revealed a radiolucent line with well-defined and well-corticated edges, uniformly separating the ossicle from the metatarsal in the right foot, consequently leading to the diagnosis of OVP. Following diagnosis, the patient was managed conservatively. Os vesalianum is not a common source of pain on the midfoot's lateral side. An oblique radiograph is an appreciable strategy to demonstrate the accessory ossicle. Thus, a careful clinical examination, coupled with the correlation of radiological findings, is a prerequisite to prevent misdiagnosis and overtreatment thereafter.

## Introduction

A well-corticated bony structure near a bone or joint, congenital in origin and resulting from unfused ossification centers, is called an accessory ossicle. These conditions may also result from post-traumatic causes or late ossification. Common locations to spot these accessory ossicles are wrist, foot, ankle, knee, hip, or shoulder. They mimic loose bodies or fractures, functioning as a conundrum leading to misdiagnosis [1]. Another theory suggests that accessory bones are rudimentary structures inherited from lower animals and frequently found during the embryonal period, which diminishes during further development [2].

Incidence of some common accessory bones, which are considered skeletal variations, is as follows: 11.7% in accessory navicular, 4.7% in os peroneum, 2.3% in os trigonum, 1.6% in os supranaviculare, 0.2% in os supratalare, and 0.2% in os intermetatarseum. Os vesalianum pedis (OVP) is among the uncommon accessory ossicles in the foot, with a low occurrence rate of about 0.1%-0.4%. It is named after Andreas Vesalius, an anatomist and physician who first described it in his study "de humani corporis fabrica" in 1543 [3-5]. The OVP is located at the proximal insertion point of the peroneus brevis tendon onto the base of the fifth metatarsal. It is important to differentiate it from the apophysis of the fifth metatarsal, which runs parallel to the shaft [1,6].

The OVP is generally an asymptomatic accessory bone, typically presenting minimal symptoms and causing no significant problems. In some cases, overuse or repetitive stress, poor biomechanics, friction and irritation, or direct trauma not only contributes to but also worsens underlying conditions, leading to the development of symptoms [1,5]. In cases of pain, a thorough differential diagnosis should be conducted to rule out other potential causes, such as a fracture at the base of the fifth metatarsal or apophysitis and an inflammatory response at the apophysis of the fifth metatarsal base [1]. Recognizing these ossicles is crucial, as it helps avoid misinterpreting them as fractures when assessing foot pain, a common clinical scenario that can lead to incorrect diagnoses and inappropriate treatment [1,4].

These tarsus anomalies can develop due to congenital factors, which may or may not be inherited. These anomalies can manifest unilaterally or bilaterally and may be susceptible to pathological alterations, highlighting the importance of awareness and monitoring [2,4]. Although most accessory ossicles and sesamoid bones in the ankle and foot do not cause symptoms, they have garnered significant attention in research due to their potential to trigger painful conditions or degenerative changes when subjected to excessive stress, overuse, or traumatic injury [3]. The location of the patient's complaints typically corresponds to the location of the accessory bones, making this the most crucial indicator in clinical evaluation. Therefore, clinicians need to possess a fundamental understanding of the typical locations of accessory bones to accurately diagnose and manage related conditions [5]. In situations where the diagnosis is challenging or underlying pathology is suspected, higher level imaging modalities like CT, MRI, and scintigraphy may provide further clarity and accuracy [1].

Only a handful of symptomatic OVP cases have been reported to date. As most OVP cases are asymptomatic, understanding the bone's role in foot pain and pathology is challenging. Only a few patients experience symptoms, particularly lateral foot pain. The limited information makes it difficult for clinicians to diagnose and manage OVP-related conditions effectively. We report a rare case of symptomatic OVP, one of the few treated conservatively.

## Case presentation

A 36-year-old male athlete in good health, a professional badminton player who recently took part in a local tournament with improper footwear, presented to the sports medicine OPD of a tertiary care center in Kancheepuram with complaints of pain on the lateral side of the right foot. The pain was insidious in onset, mild, and intermittent for six months, which resolved by itself on rest. When enquired about a history of trauma, he reported recurrent incidents of stress injuries and microtrauma during playing in the past years. In the last two months, there was aggravation in complaints as the nature of the pain was continuous, and the intensity progressed from mild to severe at the fifth metatarsal base.

The visual analog scale (VAS) score was 9 with activity and 5 on rest when the patient presented to the OPD. On physical examination, foot callosities were seen in both feet. A small swelling with tenderness at the fifth metatarsal base was observed on the right foot. The pain was provoked by turning inward and pointing downward of the right foot. Passive extension and flexion of the fifth metatarsophalangeal joint were also painful. There was no marked difference in the range of movements and stability of the ankle joint. No forefoot, midfoot, or hindfoot deformities were found, and no neurovascular deficit was found in either foot. The score of foot and ankle disability index was found to be 52, and the foot function index was 76% in the right foot.

**Figure 1:**
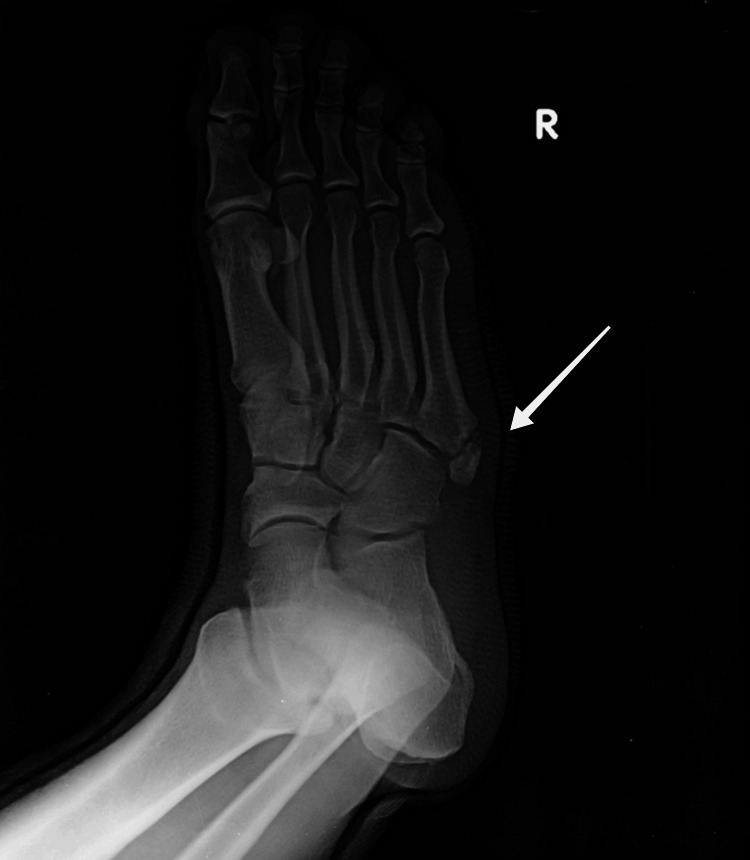
Right foot oblique radiograph. The white arrow points at OVP proximal to the fifth metatarsal base. The differential for a bony opacity in this region is an unfused apophysis orientated parallel to the cortex of the metatarsal base OVP: os vesalianum pedis

**Figure 2:**
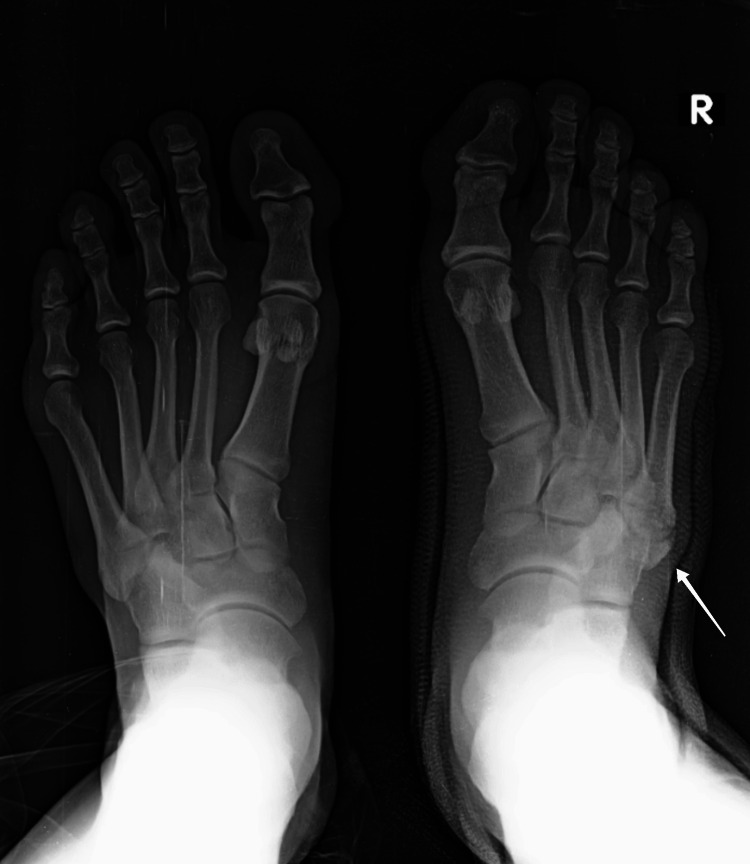
Bilateral foot anterior view radiograph. The white arrow points to OVP present in the base of the fifth metatarsal of the right foot only OVP: os vesalianum pedis

A plain anteroposterior and oblique radiographic examination of the right foot was advised, which revealed a bean-shaped oval fragment with the longest dimension being 2.8 cm at the fifth metatarsal base. Initially, this bone fragment was considered an intra-articular avulsion fracture of the fifth metatarsal base (pseudo-Jones fracture). Awareness and potential concern for OVP arose due to the absence of weight-bearing deficits in the patient who walked into the OPD without support. Thus, imaging of the contralateral foot (left) was performed, revealing normal findings. On careful reexamination of the X-ray of the right foot, a well-defined and well-corticated edged uniform radiolucent line separated the ossicle from the metatarsal rather than transverse discontinuity at the base of the fifth metatarsal. This confirmed the existence of an ossicle at the fifth metatarsal base, and it was diagnosed to be OVP (Figures 1, 2).

A fiber cast was applied above the right ankle to stabilize and restrict foot mobility. Complete activity restriction was advised, along with analgesics for pain management. The patient was also started on collagen supplements twice a day for six weeks for ligament strengthening and vitamin D 60,000 IU/week for six weeks. On review after three weeks, the cast was removed, and complete remittance of right foot pain was achieved. The VAS score in the third week was 0 on rest and 2 on activity. A reassessment of the foot and ankle disability index score was done. It was found to be 101 points, and the foot function index improved to 4%. The isometric exercise was started in the third week and was advised to be continued for a week. On the second review after a week (fourth week), the VAS score was found to be 0 on performing activity along with foot and ankle disability index of 104 and foot function index of 0%. The patient was advised to do ankle range of motion exercises, intrinsic foot muscle and gastroscopes strengthening, knee and hip strengthening, and core strengthening exercises for four weeks. Regular activities were initiated at four weeks, and the player returned to play at eight weeks.

## Discussion

To have a clear understanding of the bones and clinical features to avoid incorrect diagnosis and unnecessary intervention, Coskun et al. did a retrospective study in Turkey with 984 cases, which revealed 10.9% and 10.2% of the female and male patients, respectively, had accessory ossicles. The bilateral presence of accessory ossicles was seen in 7.6%, and right and left unilateral ossicles were seen in 7.3% and 6.3%, respectively [1,3]. Arslan et al. assessed the types of bones, both sesamoid and accessory bones of the foot, their mean size, and patterns. It was observed that among the rare accessory bones, OVP was found to be at the attachment of the peroneus brevis tendon to the base of the fifth metatarsal, with a mean size of 3.9 ± 0.8 (2.5-5.3) [7]. In our study, a bean-shaped oval bony fragment with well-corticated edges was seen in the right foot, identified as an ossicle separated from the fifth metatarsal base by a consistently spread radiolucent line. Kalbouneh et al., in their study, stated two anatomical variants of OVP: the first type, where the OVP only measures 2-4 mm in size, is located near the apex of the tuberosity of the fifth metatarsal bone, and the second type occurs when the OVP measures 10-20 mm in size, oblong-shaped with well-corticated edges. A consistently spread radiolucent line separates it from the metatarsal and cuboid bone [8]. Our study's radiological findings correlated with the second type. Beil et al., in their study, explained that OVP, a true accessory bone, is surrounded by a cortical shell. Thus, it can be differentiated from a proximal fifth metatarsal fracture, which reveals a fracture line running across or at an angle to the long axis of the metatarsal shaft, disrupting its cortical integrity [6]. Thus, with these observations in mind, with high suspicion of OVP, an X-ray of the contralateral foot was advised and reassessed. Though the diagnosis was difficult, with clinical history and the distinguishing radiological features, it was diagnosed as OVP. Some other conditions that may cause these symptoms include fractures (avulsion fracture, Jones fracture, and stress fracture), bony avulsions, incompletely ossified apophysis or residuals of apophysis (Iselin’s disease), pseudoarthritis of the base of the fifth metatarsal, and a distal avulsion of the peroneus brevis or peroneus tertius tendon, as well as lateral plantar fasciopathy [9]. Besides considering phylogenetic, ontogenetic, and posttraumatic origins, distinguishing true accessory bones from degenerative changes and ossifications along tendons and joint capsules is crucial [2,9].

In most cases, accessory bones are found to be asymptomatic. However, they can become symptomatic due to infection, degenerative disease, trauma, osteonecrosis, and other pathologies, leading to a painful condition [10]. It is also wise to consider other diagnoses like fracture and inflammation in instances of pain [1]. The classical signs our patient presented with were tenderness at the base of the fifth metatarsal with painful inversion, plantar flexion of the right foot, and passive extension and flexion of the fifth metatarsophalangeal joint with no deformities or difference in range of movements and neurovascular. This was similar to the complaints presented by Beil et al. in a 19-year-old girl [6]. Though the reasons why an asymptomatic OVP may become symptomatic or the factors contributing to the development of pain are not well understood, there are few studies that state that direct acute trauma is the reason for the OVP to become symptomatic where the pain started to be mild in nature subsiding on rest, thus gradually increasing in intensity with activity to continuous pain in athletes [9,11]. Similar progress was observed in our study, and it suggests that chronic inflammation resulting from synchondrosis joint instability due to repeated microtrauma or acute trauma may lead to the onset of pain. Other documented cases of symptoms were associated with repetitive microtrauma and persistent symptoms over time [11,12].

The previous literature states that the symptomatic OVP should initially be treated conservatively with traditional first-line methods like specialized shoe insoles, exercise routines focused on stretching, nonsteroid anti-inflammatory medications with the restricted bearing of weight, and/or casts with rest or activity modification [6,13,14]. The conservative management was very effective in our patient. However, there were many articles highlighting the limitations of conservative therapy, even in cases of full patient compliance or for recurring issues. Consequently, there has been a transition from non-surgical approaches to surgical interventions, including the straightforward removal of the ossicle from the peroneus brevis tendon through a longitudinal incision, followed by reconstruction using a tendon-to-tendon technique [6]. There were only two articles that stated a professional soccer player successfully completed seven and a half weeks of rehabilitation and returned to play with conservative treatment only and a 62-year-old male made a full recovery with conservative treatment [15]. This was similar to our player, who returned to play after eight weeks with three weeks of rest and five weeks of rehabilitation. If conservative treatment is unsuccessful, surgical options include removing a symptomatic OVP, reattaching the peroneus brevis tendon to the base of the fifth metatarsal, excising the symptomatic accessory bone without disturbing the insertion of the peroneus brevis tendon, and performing osteosynthesis with bone grafting as needed [9,12,16].

## Conclusions

OVP is commonly perceived to be a fracture of the fifth metatarsal. Though OVP is a rare case scenario, the possibility of occurrence must be considered. A high level of suspicion is necessary to achieve an accurate diagnosis. A lateral and oblique radiograph is regarded as the most effective method for visualizing the accessory ossicle and its articulation to exclude other differential diagnoses like avulsion fractures, Jones fractures, stress fractures, Iselin's disease, and additional accessory ossicles. The patient is managed conservatively, and surgery is advised in case of refractory cases. A careful clinical examination and correlation with radiological findings are a prerequisite to avoid wrong diagnosis and subsequent overtreatment.

## References

[1] Vora BM, Wong BS (2018). Common accessory ossicles of the foot: imaging features, pitfalls and associated pathology. Singapore Med J.

[2] OʼRahilly R (1953). A survey of carpal and tarsal anomalies. J Bone Joint Surg Am.

[3] Coskun N, Yuksel M, Cevener M (2009). Incidence of accessory ossicles and sesamoid bones in the feet: a radiographic study of the Turkish subjects. Surg Radiol Anat.

[4] Candan B, Torun E, Dikici R (2022). The prevalence of accessory ossicles, sesamoid bones, and biphalangism of the foot and ankle: a radiographic study. Foot Ankle Orthop.

[5] Cilli F, Akçaoğlu M (2005). The incidence of accessory bones of the foot and their clinical significance. [Article in Turkish]. Acta Orthop Traumatol Turc.

[6] Beil FT, Burghardt RD, Strahl A, Ruether W, Niemeier A (2017). Symptomatic os vesalianum: a case report and review of the literature. J Am Podiatr Med Assoc.

[7] Arslan S, Bakdik S, Oncu F, Karahan A, Durmaz M, Ozen K, Cicekbasi AE (2018). Incidence and anatomical variability of accessory and sesamoid bones of the foot. Ann Med Res.

[8] Kalbouneh H, Alajoulin O, Shawaqfeh J (2022). The anatomical variations of the lateral sesamoid bones of the foot: a retrospective radiographic analysis. Folia Morphol (Warsz).

[9] Debnar M, Kopp L, Baba V, Rammelt S (2023). Accessory bones at the foot and ankle: a comprehensive review. Fuß Sprunggelenk.

[10] Keles-Celik N, Kose O, Sekerci R, Aytac G, Turan A, Güler F (2017). Accessory ossicles of the foot and ankle: disorders and a review of the literature. Cureus.

[11] Aykanat F, Vincenten C, Cankus MC, Kose O, Sindel M (2019). Lateral foot pain due to os vesalianum pedis in a young football player; a case report and review of the current literature. Skeletal Radiol.

[12] Mousafeiris VK, Papaioannou I, Kalyva N, Arachoviti C, Repantis T (2021). Os vesalianum pedis in a young adult: a case report and literature review. Cureus.

[13] Wilson TC, Wilson RC, Ouzounov KG (2011). The symptomatic os vesalianum as an uncommon cause of lateral foot pain: a case report. J Am Podiatr Med Assoc.

[14] Petrera M, Dwyer T, Ogilvie-Harris DJ (2013). A rare cause of foot pain with golf swing: symptomatic os vesalianum pedis-a case report. Sports Health.

[15] Cruz M, Bolgla L, Alexander R, Symbas P, Royal J, Serrano-Dennis J (2023). Conservative treatment of a symptomatic os vesalianum pedis in a professional soccer player: a case report. Internet J Allied Health Sci Pract.

[16] Rammelt S, Neumann AP (2022). Resection of a symptomatic os vesalianum with peroneus brevis reattachment: a report of 3 cases and literature review of 100 years. Foot Ankle Orthop.

